# Jiyuan oridonin A induces differentiation of acute myeloid leukemia cells including leukemic stem-like cells

**DOI:** 10.3389/fphar.2022.1001552

**Published:** 2022-09-05

**Authors:** Fahui Li, Congying Gao, Xueming Li, Jiangyun Wang, Yao Zhao, Yu Ke, Ying Liu, Hong-Min Liu, Zhenbo Hu, Liuya Wei, Zhe-Sheng Chen

**Affiliations:** ^1^ School of Pharmacy, Weifang Medical University, Weifang, China; ^2^ Laboratory for Stem Cell and Regenerative Medicine, Affiliated Hospital of Weifang Medical University, Weifang, China; ^3^ School of Pharmacy, Zhengzhou University, Zhengzhou, China; ^4^ Department of Pharmaceutical Sciences, College of Pharmacy and Health Sciences, St. John’s University, Queens, NY, United States

**Keywords:** acute myeloid leukemia, leukemia stem cells, differentiation blockade, cytarabine chemotherapy resistance, refractory/relapsed AML

## Abstract

Acute myeloid leukemia (AML) is an aggressive form of hematological neoplasia characterized by failure of myeloid differentiation. AML is a leading cause of death from leukemia. Cytarabine chemotherapy resistance is a major source of refractory/relapsed AML. A major obstacle to the successful treatment of AML results from residual disease maintained by leukemic stem cells (LSCs), which are mostly resistant to conventional chemotherapy. Here, we determined the effect of a natural compound, Jiyuan oridonin A (JOA), on the differentiation blockade in the M2 subtype [particularly t (8;21)] of AML cells, M3 subtype of AML cells (APL cells), and leukemic stem-like cells both *in vitro* and *in vivo*. We found that JOA induced cell differentiation and suppressed the colony formation capacity in various AML cell lines (Kasumi-1, KG-1, MUTZ-8, NB4, and HL-60) without eliciting apoptosis. The mechanism of JOA-induced cell differentiation depends on the specificity of cell type. JOA mediated the differentiation of Kasumi-1 cells by activating the hematopoietic cell lineage signaling pathway, while inhibition of c-MYC was involved in the JOA-induced differentiation of NB4 cells. Moreover, JOA was identified to target leukemic stem-like cells by induced cell differentiation *in vivo*. These findings demonstrated that JOA could inhibit the proliferation of M2 and M3 subtypes of AML cells and leukemic stem-like cells by overcoming the differentiation blockade, which may offer a novel therapeutic strategy for AML to overcome relapse and drug resistance in patients with AML. Our findings highlight the possibility of using compounds like JOA as a promising differentiation-induced agent for the treatment of AML.

## Introduction

Leukemia is a malignant clonal stem cell neoplasm with the accumulation of immature hematopoietic cells. It is characterized by the block of differentiation and unrestricted rapid cell proliferation (Fujita et al., 2018). Acute myeloid leukemia (AML) is an aggressive type of leukemia with a block in myeloid cell differentiation, which is responsible for a majority of death cases from leukemia (Bonnet and Dick, 1997a). In the past 50 years, a combination of cytarabine (Ara-C) and anthracyclines has been used as the standard of care for leukemia (Assouline et al., 2012). However, whereas 70–80% of AML patients achieve remission, following completion of induction chemotherapy, and 50% of them relapse, and there is no salvage regimen existing currently (Ishikawa et al., 2007a). Cytarabine chemotherapy resistance is a major source of refractory/relapsed AML (Chaudhuri et al., 2013). Hence, the biggest challenge in the treatment of AML is the refractory disease and relapse after having achieved a complete remission. The t(8;21) is one of the most frequent chromosomal translocations in AML, observed in 12–20% of all cases of AML and 40–80% of the M2 subtype AML (Zhou et al., 2007). Patients with t(8;21) AML treated with the cytarabine-based protocol are considered to have a good prognosis; however, approximately 50% of them relapse (Yu et al., 2016; Li et al., 2021). Therefore, the development of novel agents is needed for alternative therapy for t(8;21) AML and to improve patient outcomes. In addition, acute promyelocytic leukemia (APL), the M3 subtype of AML, represents 10–15% of the cases of AML in adults (Soignet et al., 1998). APL is one of the most aggressive and fatal forms of acute leukemia with poor outcomes (Rao et al., 2013). A differentiation inducer, all-trans retinoic acid (ATRA), has transformed APL from a highly fatal disease to now the most frequently curable acute leukemia (Huang et al., 1988; Wang and Chen, 2008). However, ATRA is not effective in other types of AML. Furthermore, it is known that the outcome of leukemia depends on a small population of “leukemic stem cells” (LSCs). LSCs are involved in the initiation, drug resistance, and relapse of leukemia (Bonnet and Dick, 1997b; Ishikawa et al., 2007b). LSCs in leukemia are defined as CD34^+^CD38^−^primitive progenitor cells. These cells have self-renewal properties and suppressed the differentiation ability (Sachlos et al., 2012). Moreover, LSCs are mostly resistant to conventional chemotherapy. Their relatively static or dormant state and location in a protective bone marrow niche are some of the critical components contributing to chemotherapy resistance. The eradication of LSCs is an unmet clinical need and may be achieved by overcoming the differentiation block of LSCs.

Jiyuan oridonin A (JOA) is a small molecule of ent-kaurene diterpenoid isolated from *Isodon rubescens* (Ke et al., 2018). Our previous work had shown that JOA inhibited the proliferation of AML cells with mixed lineage leukemia gene rearrangements by promoting cell differentiation *in vitro* (Qu et al., 2021). Herein, the present work investigated the efficacy of JOA on the differentiation block of the M2 subtype of AML cells [particularly (8;21)], M3 subtype of AML cells (APL cells), and leukemic stem-like cells both *in vitro* and *in vivo*. Additionally, we also explored the possible molecular mechanism involved in the JOA-induced cell differentiation. This study reveals the role of JOA in overcoming the differentiation block of AML cells, particularly leukemic stem-like cells.

## Materials and methods

### Chemicals

**FIGURE 1:**
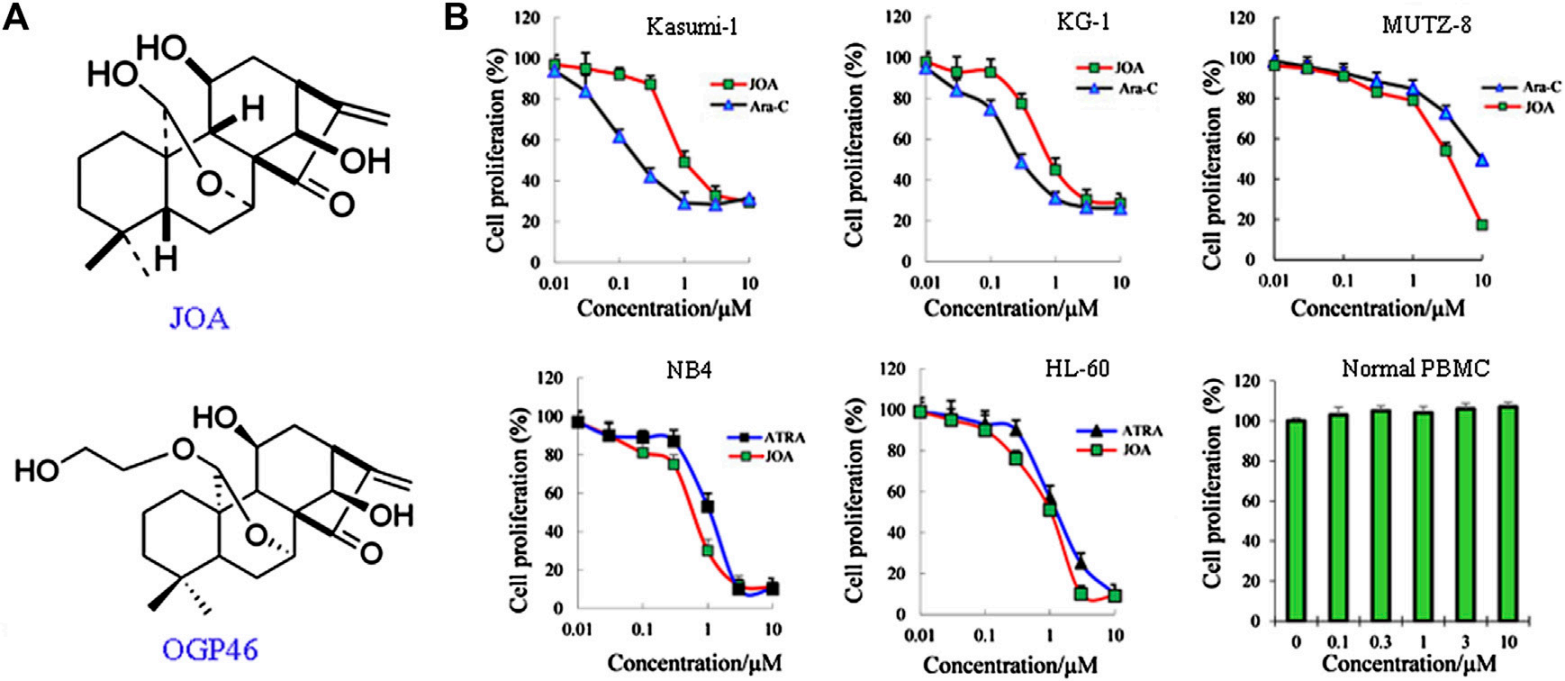
JOA inhibited cell proliferation of Kasumi-1, KG-1, MUTZ-8, NB4, HL-60 cells, and human normal peripheral blood mononuclear cells. **(A)** Chemical structure of JOA and OGP46. **(B)** Effect of JOA, Ara-C, or ATRA in the proliferation of cells at 72 h. The points with error bars represent the mean ± SD. Figures are representative of three independent experiments performed in triplicates.

The chemical structure of JOA and its analog OGP46 (Zhao et al., 2021) is shown in Figure 1A. The molecular weight of JOA is 348.2. ATRA and Ara-C were purchased from Sigma-Aldrich (St. Louis, MO, United States). RPMI-1640, fetal bovine serum (FBS), 5,000 units/mL of penicillin, and 5,000 μg/mL of streptomycin were purchased from Gibco (Carlsbad, CA, United States). Propidium iodide (PI)/RNase staining buffer and the Annexin Ⅴ/PI Apoptosis Detection Kit were purchased from BD biosciences (San Jose, CA, United States). 3-(4, 5-Dimethylthiazol-2-yl)-2,5-diphenyltetrazolium bromide (MTT) was obtained from Solarbio (Beijing, China). FITC anti-CD11b (cat #301403), FITC anti-CD14 (cat #301804), FITC anti-CD64 ((cat #399505), PE anti-CD13 (cat #301704), and PE anti-CD15 (cat #301906) were obtained from Biolegend Inc. (San Diego, CA, United States). PE anti-human HLA-DR (FAB4869P) was purchased from R&D systems (Minneapolis, MN, United States). Anti-human HLA-DP (sc-33719) was obtained from Santa Cruz Biotechnology, Inc. (Dallas, TX, United States). Anti-human CD38 (Cat# 345807) and PE anti-human CD34 (Cat# 550619) antibodies were purchased from BD Pharmingen (San Diego, CA, United States). MethoCult H4100 (Cat# 04100) and H4435 (Cat# 04435) were purchased from STEMCELL Technologies (Vancouver, BC, Canada). Monoclonal antibodies against GAPDH (cat# 5,174) and antibodies against CDKN2A (cat# 92803) and c-MYC (Cat #9402) were purchased from Cell Signaling Technology Inc. (Beverly, MA, United States). Antibodies against CD64 (ab134073), G-CSFR (ab233833), and CCND1 (ab134175) were purchased from Abcam (Cambridge, MA). The PrimerScript^TM^ RT reagent kit and the SYBR Premix Ex Taq™ reagent kit were purchased from TaKaRa Bio Inc. (Otsu, Japan). All flow cytometry analyses were conducted using a FACSCalibur System (BD Biosciences, San Diego, California, United States). PCR amplification was performed using an Applied Biosystems 7500 Fast Real-Time PCR System (Thermo Fisher Scientific Inc., Waltham, United States).

### Cell lines and cell culture

Kasumi-1 (the M2 subtype of AML with t(8;21) translocation, DSMZ: ACC 220), KG-1 (leukemic stem-like cells established from the bone marrow cells of an AML patient, characterization of 98% CD34^+^ cells, DSMZ: ACC 14), MUTZ-8 (leukemic stem-like cells, characterization of 84% CD34^+^ cells, ACC 689) (Hu et al., 2002), NB4 (M3 subtype of AML, APL cell line, DSMZ: ACC 207), and HL-60 (the M2 subtype of AML, ACC 3) cell lines were used. Cord blood samples from three healthy individuals (obtained from the Affiliated Hospital of Weifang Medical University, Weifang, China) were collected after obtaining written informed consent from the donors. Mononuclear cells were isolated using the Histopaque 1077 solution. All cells were cultured at 37°C, in 5% CO_2_ with RPMI 1640, supplemented with 10% FBS and 1% penicillin/streptomycin.

### Cell proliferation assay

The effect of JOA on cell proliferation compared with Ara-C or ATRA were determined using a modified MTT colorimetric assay. About 5 × 10^3^ cells were seeded into 96-well plates. After incubation with JOA, Ara-C, or ATRA for 72 h, 20 μL of MTT (4 mg/mL) was added to each well, and the cells were further incubated at 37°C for 4 h. Following incubation, the plates were centrifuged, and the formazan crystals were dissolved in 100 μL of DMSO. The light absorbance was measured at 570 nm using an Opsys microplate reader (Dynex Technologies, United States).

### Cell cycle analysis

Kasumi-1, KG-1, MUTZ-8, NB4, and HL-60 cell lines were incubated with the indicated concentration of JOA for 24, 48, or 72 h. The cells were collected, fixed, and stained with 50 μg/mL of propidium iodide (PI) containing 100 μg/mL RNase A. Flow cytometric analysis was used to determine the percentage of cells in different phases of the cell cycle. The data were analyzed by FlowJo 7.6.1 software.

### Colony formation assay

About 5 × 10^3^ cells (Kasumi-1, KG-1, MUTZ-8, NB4, and HL-60 cell lines) were cultured with the indicated concentration of JOA in 500 μL of 2.6% methylcellulose medium containing 10% FBS in 24-well plates. After incubation for 14 days, the number of separate colonies was counted under a macroscope.

### Apoptosis analysis

Cells were incubated with indicated concentrations of JOA, Ara-C, or ATRA for 72 h. The cells were collected, washed with PBS, and incubated with FITC-labeled annexin-V and PI at 37°C for 30 min. Flow cytometric analysis was performed to determine the apoptotic cell population using a BD FACSCalibur System.

### Cell morphology analysis

Cells were incubated with the indicated concentration of JOA and collected for analysis. Cytospin smears were prepared, and the cells were stained with Wright–Giemsa for 15 min. The morphological features of cells were observed under a light microscope (1,000 × magnification).

### Cell differentiation assessment

Cells were cultured with the indicated concentration of JOA. After incubation, cells were collected and incubated with their respective monoclonal antibodies for 30 min at 4°C. The following cell surface antigens were used to assess cell differentiation: monocyte/granulocyte biomarkers (CD11b, CD13, CD14, and CD15), monocyte/macrophage biomarkers (CD64), immune regulation antigens (HLA-DR and HLA-DP), CD34, and CD38. The results are expressed as the mean fluorescence intensity of 10,000 cells.

### mRNA sequencing

mRNA sequencing was performed in Kasumi-1 and NB4 cell lines by the Xiuyue Biotechnology Co., Ltd., Shandong, China. Briefly, RNA was extracted, and the quality was assessed. RNA was purified and converted to an Illumina sequencing library, and the library was validated. The expressions of mRNAs were estimated using transcripts per million (TPM). The differential expression test was analyzed using DESeq R packages, according to the package manual. The corrected *p*-value of 0.05 and the absolute value of log_2_ FC (fold change) ≥ 0.58 were set as a threshold for the differential expression of genes (DEGs). Kyoto Encyclopedia of Genes and Genomes (KEGG) pathway enrichment analysis was used to understand the signaling pathway enrichment of DEGs for the Kasumi-1 cell line. Gene Set Enrichment Analysis (GSEA) was conducted, and C2 curated functional gene sets from the Molecular Signature Database were used to analyze the potential signaling pathways involved in the NB4 cell line. *p* < 0.05 was set as the significance threshold.

### Quantitative real-time PCR

Total RNA was extracted using the TRIzol® reagent (Invitrogen Life Technologies). The cDNA was transcribed from 1 μg of total RNA using a PrimerScript^TM^ RT reagent kit with a gDNA Eraser. Quantitative, real-time PCR was performed in triplicate using SYBR Green I and hotstart Taq DNA polymerase in an Applied Biosystems 7500 Fast Real-Time PCR System. The relative quantification of gene expression was calculated using the 2^−ΔΔCt^ method. The following primers are used as follows: GAPDH (forward 5´-TGG​GTG​TGA​ACC​ATG​AGA​AGT-3´ and reverse GAPDH 5´-TGA​GTC​CTT​CCA​CGA​TAC​CAA-3´), CD64 (forward 5´-GAA​GGG​GTG​CAC​CGG​AAG​G-3´ and reverse 5´-CAC​GGG​GAG​CAA​GTG​GGC​AG-3´), HLA-DPA1 (forward 5´-TGT​AAA​ACG​ACG​GCC​AGT​ACA​TTT​TGT​CGT​GTT​TTT​CTC​T-3´ and reverse 5´-CAG​GAA​ACG​GCT​ATG​ACC​CTC​TCA​TCC​CTT​CCA​GTT​G-3), CDKN2A (forward 5´-GCT​TCT​CAC​CTC​GCT​TGT​CA-3´ and reverse 5´-AGT​GAC​CAA​GAA​CCT​GCG​AC-3´), c-MYC (forward 5´- CAG​CTG​CTT​AGA​CGC​TGG​ATT​T-3´ and reverse 5´- ACC​GAG​TCG​TAG​TCG​AGG​TCA​T-3´), G-CSFR (forward 5´- CTG​GAG​GAT​GGA​ACA​GAA​TG-3´ and reverse 5´-GAA​GAT​GGT​GAG​TGG​GTA​AGG-3´), and CCND1 (forward 5´-GAA​GAT​CGT​CGC​CAC​CTG-3´ and reverse 5´- GAC​CTC​CTC​CTC​GCA​CTT​CT-3´).

### Western blotting analysis

Total cellular proteins were isolated with lysis buffer. Equal amounts of protein were subjected to SDS-PAGE and transferred onto polyvinylidene fluJOAe (PVDF) membranes. The membranes were blocked and then incubated with antibodies. Images were obtained using an enhanced chemiluminescence reagent (ECL) detection system (Amersham Imager 600, GE Healthcare Biosciences, Pittsburg PA).

### MUTZ-8 cell xenograft in NOD/SCID mice

We next evaluated the activity of JOA against MUTZ-8 cells in a tumor xenograft model using NOD/SCID mice (Weitong Lihua Experimental Animal Technology Co. Ltd., Beijing, China). MUTZ-8 cells (1 × 10^7^) were inoculated *via* tail vein into sub lethally irradiated NOD/SCID mice in a final volume of 0.2 mL of PBS with 0.5% FBS. After 4 weeks, the successful engraftment of human cells (CD34^+^) in peripheral blood was detected by flow cytometry, and then, these mice were randomly assigned into four groups (six mice in each group) and treated for 7 days with an interval of 1 day with control (PBS, i. v.) or JOA (20 mg/kg/d, 30 mg/kg/d, i. v.) or Ara-C (25 mg/kg/d, i. v.). Disease evolution and effect of drugs on LSC-enriched compartments were determined after 7 days of treatment by flow cytometric analysis of human CD11b, CD15, and CD34^+^CD38^−^ cells in the bone marrow, and the engraftments were analyzed by staining of bone marrow cells. Furthermore, 20,000 mononuclear cells of the bone marrow from the four groups of mice were cultured in 500 μL of 2.6% methylcellulose medium (H4435, stem cell) containing 10% FBS in 24-well plates for 14 days, and then, the colony formation was detected by light microscopy.

### Statistical analysis

All experiments were repeated three times unless otherwise stated. The results are expressed as the mean ± standard deviation (SD). The difference between the mean of each experimental group with the control was analyzed by the one-way ANOVA method by SPSS software, and *p* < 0.05 was considered statistically significant.

## Results

### Jiyuan oridonin A significantly inhibits the proliferation of acute myeloid leukemia cells

As shown in Figure 1B, JOA significantly reduced the proliferation of Kasumi-1, KG-1, and MUTZ-8 cells with the IC_50_ values of 0.98, 0.89, and 3.75 μM, which was comparable with those of 0.22, 0.29, and 9.53 μM of Ara-C, respectively. Additionally, JOA significantly inhibited the proliferation of NB4 and HL-60 cells with the IC_50_ value of 0.68 and 1.05 μM as compared with ATRA (IC_50_ is 1.14 and 1.43 μM, respectively). Importantly, JOA was 2.5-fold more potent than Ara-C in inhibiting the proliferation of MUTZ-8 cells *in vitro*. This result suggests that JOA is efficacious in decreasing the proliferation of the M2 subtype of AML cells including the t(8,21) AML cells, APL cells, and leukemic stem-like cells, especially the MUTZ-8 cells. Notably, JOA did not have any significant effect on the proliferation of normal peripheral blood mononuclear cells (PBMCs).

### Jiyuan oridonin A induces G0/G1 cell cycle arrest in acute myeloid leukemia cells

**FIGURE 2:**
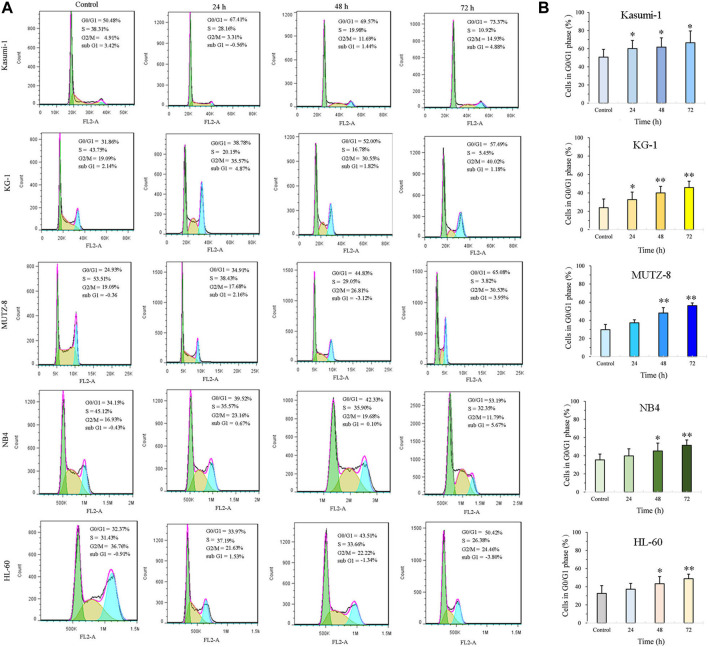
JOA induced cell cycle exit at the G0/G1 phase in Kasumi-1, KG-1, MUTZ-8, NB4, and HL-60 cells. **(A)** Effect of JOA on the cell cycle of cells. **(B)** Percentage of G0/G1 cells presented as a bar graph (*p < 0.05 and **p < 0.01). Kasumi-1, KG-1, MUTZ-8, NB4, and HL-60 cells were incubated with 1, 1, 4, 1, or 1 μM of JOA, respectively, for 24 h, 48 h, or 72 h.

JOA induces a similar constant increase in the percentage of cells at the G0/G1 phase up to 72 h in Kasumi-1, KG-1, MUTZ-8, NB4, and HL-60 cells (Figures 2A,B). These findings suggest that JOA may inhibit cell proliferation by inducing a G0/G1 cell cycle arrest.

### Jiyuan oridonin A significantly decreases colony formation in acute myeloid leukemia cells

**FIGURE 3:**
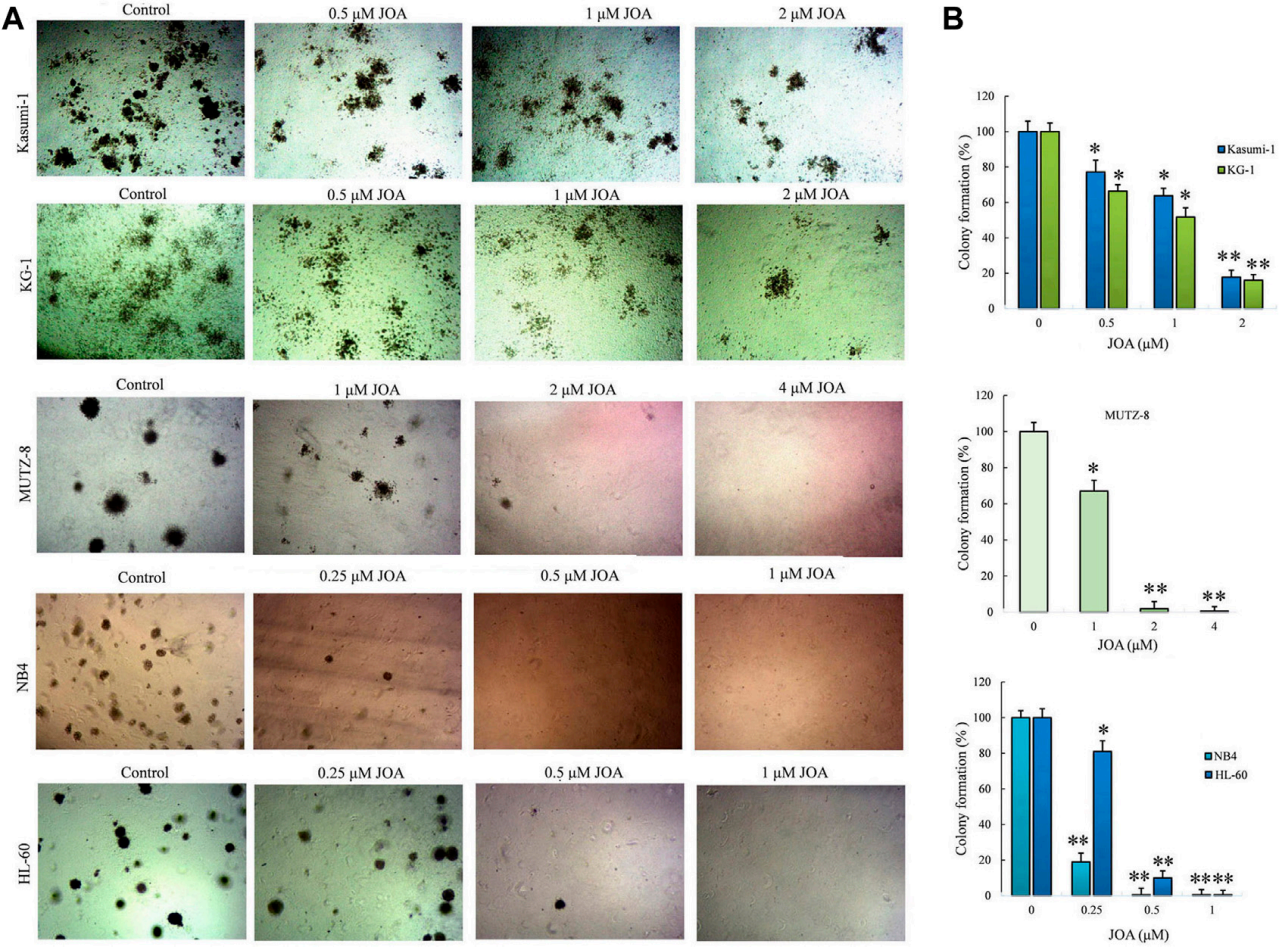
Effect of JOA on the colony formation ability in Kasumi-1, KG-1, MUTZ-8, NB4, and HL-60 cells. **(A)** Colony formation assay in methylcellulose. **(B)** Effect of different concentrations of JOA on the number of colonies (*p < 0.05 and **p < 0.01). Cells were incubated with JOA (0.25–2 µM) for 14 days.

We next determined the long-term effect of JOA on the colony-forming ability of Kasumi-1, KG-1, MUTZ-8, NB4, and HL-60 cells. It can be seen from Figures 3A,B that the number of colonies decreased with the increasing concentration of JOA. Moreover, MUTZ-8, NB4, and HL-60 cells could hardly form colonies when incubated with JOA at 2, 0.5, and 1 μM, respectively. The colony formation rate of Kasumi-1 and KG-1 cell lines is 17.8% and 26.0%, respectively, when they were treated with JOA at a concentration of 2 µM. These results show that JOA suppresses colony formation in these cells.

### Jiyuan oridonin A does not induce prominent apoptosis in acute myeloid leukemia cells

**FIGURE 4:**
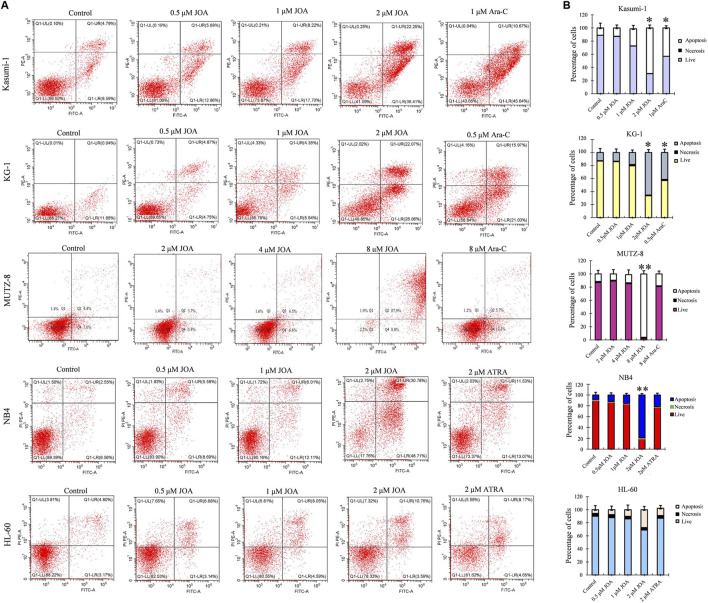
JOA induced minimal signs of apoptosis in Kasumi-1, KG-1, MUTZ-8, NB4, and HL-60 cells. **(A)** Analysis of apoptotic cells using the Annexin V/PI apoptosis detection kit. **(B)** Percentages of apoptotic cells are presented as bar graphs (*p < 0.05 and **p < 0.01). Cells were incubated with JOA (0.5–8 μM), Ara-C (0.5 or 1 μM), or ATRA (2 μM) for 72 h.

In order to determine whether the anti-proliferative activity of JOA on Kasumi-1, KG-1, MUTZ-8, NB4, and HL-60 cells was associated with the induction of apoptosis induced by JOA, these cells were treated with indicated concentrations of JOA, Ara-C, or ATRA for 72 h, and the cell apoptosis rate was determined. As shown in Figures 4A,B, Kasumi-1, KG-1, MUTZ-8, NB4, and HL-60 cells showed minimal apoptosis when cells were incubated with JOA at a concentration of lessthan 1, 1, 4, 1, or 2 μM, respectively. These results indicate that the cell cycle arrest was not associated with cell apoptosis in Kasumi-1, KG-1, MUTZ-8, NB4, and HL-60 cells treated with JOA at 1, 1, 4, 1, or 1 μM, respectively. Hence, these concentrations of JOA were chosen for the following experiment to explore its effect on cell differentiation.

### Jiyuan oridonin A induces acute myeloid leukemia cell differentiation

**FIGURE 5:**
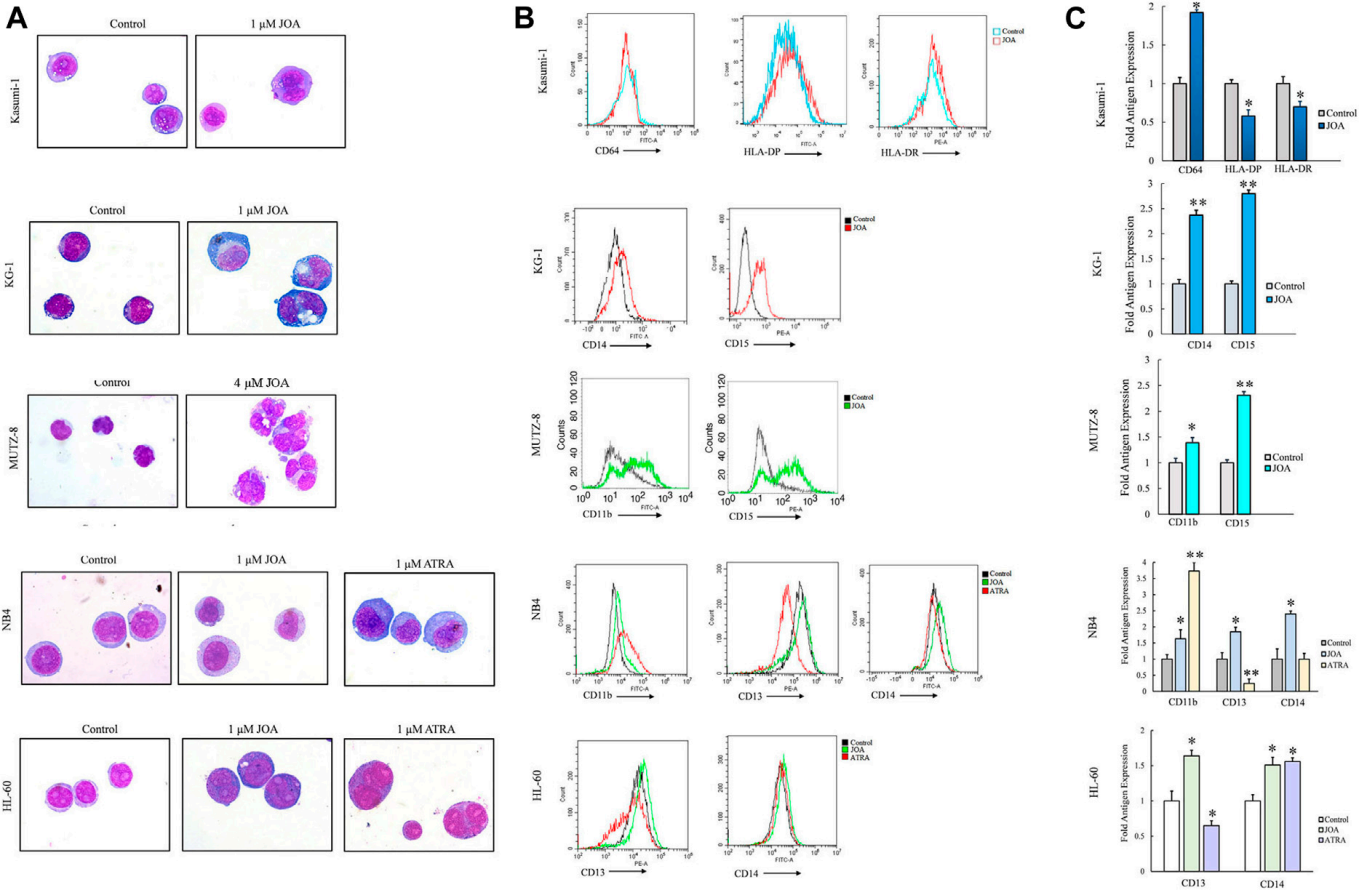
JOA induced the differentiation of Kasumi-1, KG-1, MUTZ-8, NB4, and HL-60 cells based on morphological features and cell membrane markers. **(A)** Wright–Giemsa staining on the morphological changes of cells captured by an oil immersion lens (1,000×). **(B)** Expression of cell surface antigens. **(C)** Graph bars presenting the mean fluorescence intensity of antigens (*p < 0.05 and **p < 0.01). Cells were incubated with 1, 1, 4, 1, or 1 μM of JOA, respectively for 72 h.

Since JOA inhibited the proliferation of Kasumi-1, KG-1, MUTZ-8, NB4, and HL-60 cells without inducing prominent apoptosis, we evaluated the effect of JOA on the morphological features and cell membrane markers of differentiation in these cells. As shown in Figure 5A, Kasumi-1, KG-1, MUTZ-8, NB4, and HL-60 cells all showed polyploidization and obvious phenotypic change. Moreover, it can be seen from Figures 5B,C that the expression of CD64 was increased whereas that of HLA-DP and HLA-DR was decreased in Kasumi-1 cells. JOA significantly upregulated the expression of CD14 and CD15 in KG-1 cells and increased the expression of CD11b and CD15 in MUTZ-8 cells. Similarly, the expression levels of CD13 and CD14 were increased in both NB4 and HL-60 cells, whereas CD11b was significantly upregulated in NB4 cells. These data suggest that the anti-proliferation effect of JOA in Kasumi-1, KG-1, MUTZ-8, NB4, and HL-60 cells may result from the induction of cell differentiation.

### Jiyuan oridonin A induces cell differentiation by regulating different signaling pathways in Kasumi-1 and NB4 cells

**FIGURE 6:**
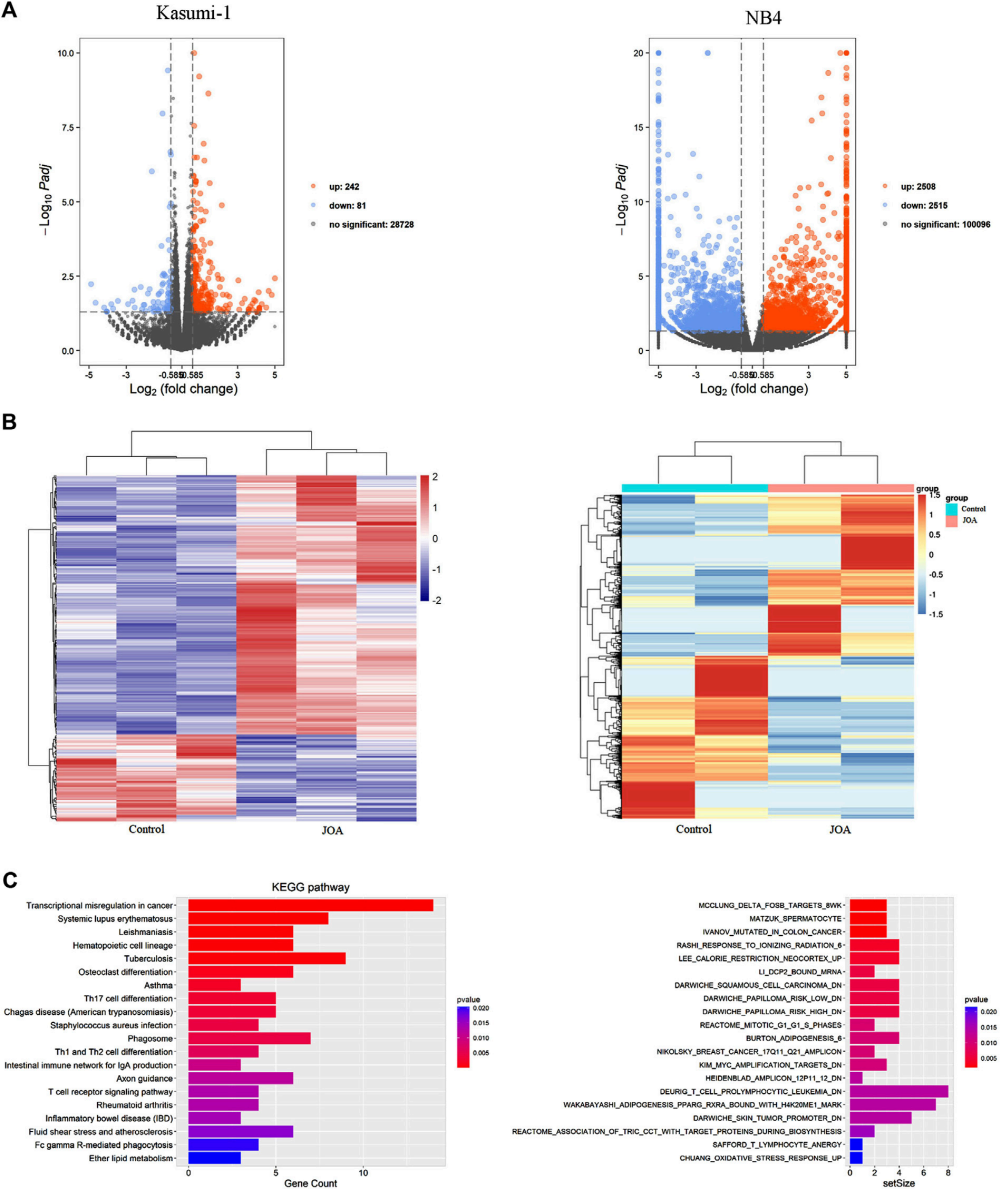
mRNA sequencing analysis in Kasumi-1 and NB4 cells treated with JOA. **(A)** Volcano plots of Kasumi-1 and NB4 cells. **(B)** Heatmap of all DEGs. The bars from blue or violet to red denote the expression levels of DEGs from low to high. **(C)** KEGG or GSEA analyses on all DEGs. Kasumi-1 and NB4 cells were incubated with 1 µM of JOA for 48 h.

To understand the mechanism of action of JOA in inducing cell differentiation, we examined the mRNA expression by mRNA-Seq in Kasumi-1 and NB4 cells. The volcanic diagram of Kasumi-1 and NB4 cells is shown in Figure 6A. A total of 242 genes were upregulated, and 81 genes were down-regulated in Kasumi-1 cells. Similarly, 2,515 genes were down-regulated, and 2,508 genes were up-regulated in NB4 cells. These data indicated that JOA is not a global gene transcription inhibitor, which is presented in Figure 6B. As shown in Figure 6C, KEGG analysis showed that the most prominent pathway associated with cell differentiation induced by JOA was the hematopoietic cell lineage signaling pathway (e.g., FCGR1B (CD64β), FCGR1CP (CD64c), HLA-DPA1, HLA-DRA, CD8A, and IL6R genes were enriched) in Kasumi-1 cells. GSEA analysis exhibited that the dominant pathway activated by JOA was the KIM_MYC_AMPLIFICATION_TARGETS_DN signaling pathway in NB4 cells. Because MYC gene amplification was correlated with the expression of MYC mRNA and protein (Holien et al., 2015), the pathway is associated with the decrease of MYC.

**FIGURE 7:**
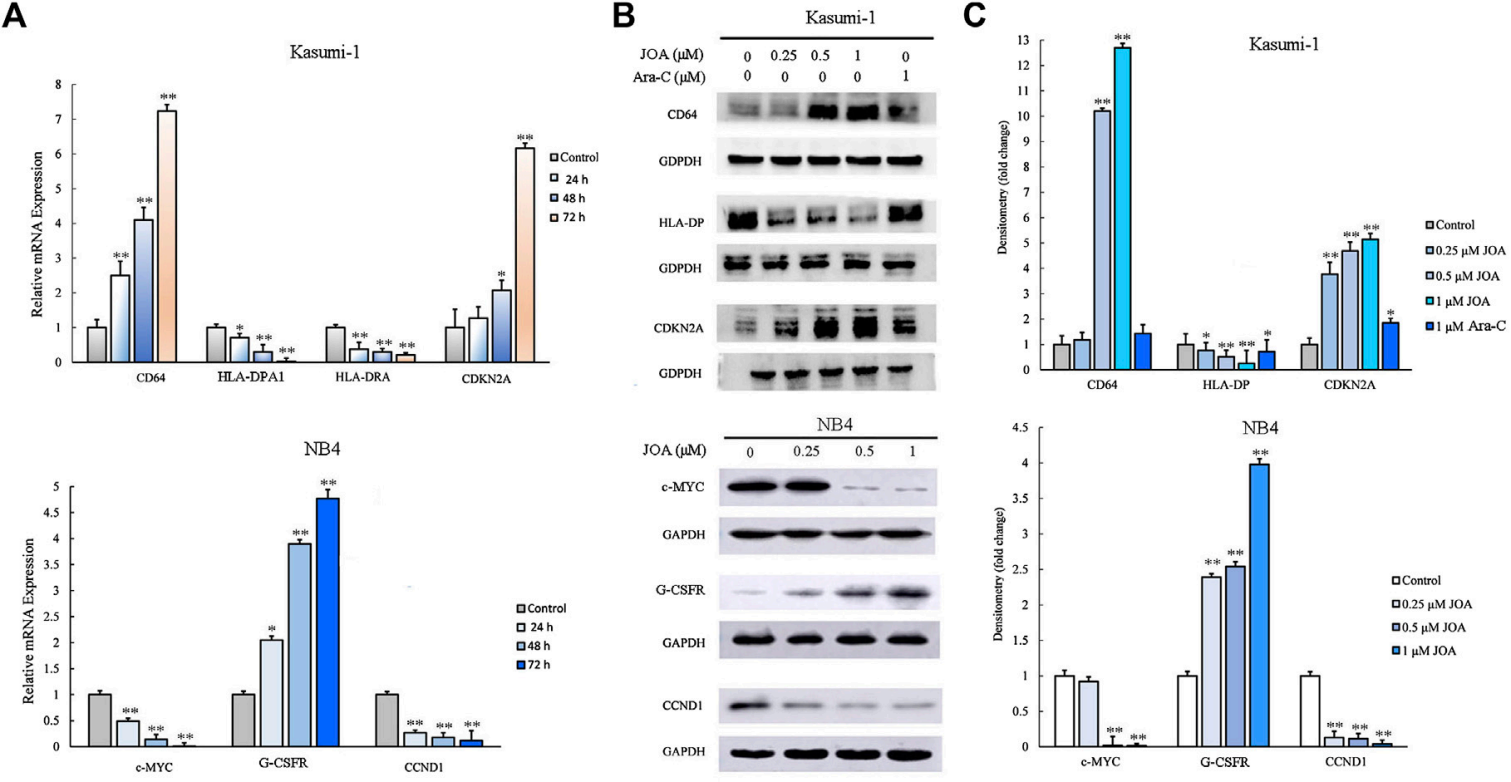
Molecular analysis of the effects of JOA on differentiation-related genes in Kasumi-1 and NB4 cells. **(A)** Effect of JOA on the mRNA expression of CD64, HLA-DPA1, HLA-DRA, and CDKN2A in Kasumi-1 cells and c-MYC, G-CSFR, and CCND1 in NB4 cells. The cells were incubated with 1 μM of JOA for 24, 48, or 72 h. **(B)** Effect of JOA on protein expression of CD64, HLA-DP, and CDKN2A in Kasumi-1 cells, and c-MYC, G-CSFR, and CCND1 in NB4 cells. The cells were incubated with JOA (0.25, 0.5, or 1 μM) or Ara-C (1 μM) for 72 h. **(C)** Graph bars showing the protein expression quantified using an AI600 imager (*p < 0.05 and **p < 0.01).

To validate the differentially expressed genes identified by mRNA-sequencing, real-time PCR and Western blotting were conducted in Kasumi-1 and NB4 cells. As shown in Figures 7A–C, the protein and mRNA expression of CD64 were significantly increased while those of HLA-DPA1 or HLA-DPA and HLA-DRA were decreased, following incubation with 1 μM of JOA in Kasumi-1 cells. In addition, the transcriptional and protein levels of cyclin-dependent kinase inhibitor 2 A (CDKN2A) were significantly increased in Kasumi-1 cells incubated with 1 μM of JOA. Similarly, c-MYC and cyclin D1 (CCDN1) were significantly downregulated whereas the granulocyte colony-stimulating factor receptor (G-CSFR) was significantly upregulated in NB4 cells treated with 1 μM of JOA.

### Jiyuan oridonin A inhibits the proliferation of leukemic stem-like cells *in Vivo*

**FIGURE 8:**
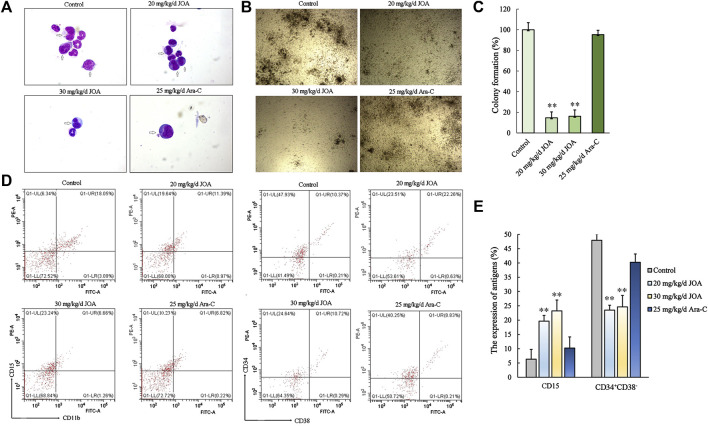
JOA inhibited the proliferation of leukemic stem-like cells in the MUTZ-8 cell xenograft in the NOD/SCID mouse model. **(A)** Bone marrow cells were stained with Wright–Giemsa staining, and the morphology of leukemia cells (marked by arrows) was observed. **(B)** Colony formation ability of mononuclear cells from the bone marrow of mice. **(C)** Graph bars showing the colony formation number (**p < 0.01). **(D)** Expression of CD11b (FITC)-CD15 (PE) and CD34^+^CD38^−^ cells in the mononuclear cell from the bone marrow of mice. The CD34^+^CD38^−^ cells were detected by flow cytometry, in which the CD34 antibody was PE-labeled and the CD38 antibody was FITC labeled. **(E)** Graph bars showing the expression of antigens (**p < 0.01).

As shown in Figures 8B,C, the colony-forming ability of bone marrow cells from the JOA treatment group (20 mg/kg/d, 30 mg/kg/d, respectively) was greatly reduced, while no significant difference was shown between the Ara-C treatment group and the control group, indicating that JOA selectively targets leukemic stem-like cell populations. This result was in accordance with the result that the ratio of CD34^+^CD38^−^ was reduced from 47.93% in the control to 23.51% and 24.64% of the JOA group (20 mg/kg/d and 30 mg/kg/d, respectively) while that of the Ara-C treatment group was 40.25% (Figures 8D,E). Moreover, it can be seen from Figure 8A that JOA induced the differentiation of AML cells with morphological changes. In addition, JOA can increase the expression of CD15 from 6.34% in the control to 19.64% and 23.24% in the JOA treatment groups (Figures 8D,E). These data demonstrate that JOA significantly reduces the proliferation of leukemic stem-like cells by inducing cell differentiation *in vivo*.

## Discussion

AML is the leading cause of death from leukemia. Ara-C has been an essential medication used in the clinical treatment of AML for the past 50 years. There is no standardized salvage regimen for AML. Most AML patients display an unsatisfactory prognosis due to the high prevalence of refractory and relapsed disease resulting from Ara-C resistance, which is the biggest challenge in the treatment of AML. Herein, we developed a novel natural ent-kaurene diterpenoid compound that can effectively surmount differentiation blockade in the M2 subtype, especially t (8;21), M3 subtype, and leukemic stem-like AML cells both *in vitro* and *in vivo*. Ent-kaurane diterpenoid compounds, such as oridonin and OGP46, exhibited anti-cancer activity, particularly for leukemia (Liu et al., 2011; Zhen et al., 2012; Huang et al., 2017; Wei et al., 2020). In this study, we found that JOA could markedly inhibit cell proliferation and colony formation by inducing the differentiation of Kasumi-1, KG-1, MUTZ-8, NB4, and HL-60 cells with morphological changes and alteration of the expression of hematopoietic differentiation antigens such as CD64, HLA-DP, HLA-DR, CD11b, CD13, CD14, or CD15. The cell differentiation was coupled with the G0/G1 cell cycle arrest. Furthermore, the activity of the anti-leukemic effect of JOA was comparable with ATRA and was better than its analogs OGP46 with a smaller IC_50_ value at 72 h than that of OGP46 at 96 h (Zhao et al., 2021). Interestingly, we found that JOA could functionally target leukemic stem-like cell populations (CD34^+^CD38^−^) by inducing cell differentiation, while Ara-C has no activity in the MUTZ-8 cell xenograft NOD/SCID mouse model. Overall, we found that JOA is effective against the M2 subtype of AML cells, especially t (8;21), the M3 subtype of AML cells, and leukemic stem-like cells by inducing cell differentiation both *in vitro* and *in vivo*.

Hematopoietic cell lineage is a differentiation-related pathway representing the development and differentiation of hematopoietic cells into various hematopoietic lineages (Zhang et al., 2017). In the present report, it was shown that the prominent signaling pathway related to cell differentiation in Kasumi-1 cells treated with JOA is the hematopoietic cell lineage signaling pathway, in which *CD64β*, *CD64c*, *HLA-DPA1*, *HLA-DRA*, *CD8A*, and *IL6R* genes were enriched. Moreover, JOA treatment promoted cell differentiation with morphological changes and with an increase in the expression of the cell surface antigen CD64 and decreased the expression levels of HLA-DP and HLA-DRA in Kasumi-1 cells. These findings indicate that JOA-mediated differentiation of Kasumi-1 cells could be achieved by activating the hematopoietic cell lineage signaling pathway and changing the expression of cell surface antigens.

It has been revealed that impairment of cell differentiation plays an important role in c-MYC-induced tumorigenesis (Leon et al., 2009; Abraham et al., 2016; Bahr et al., 2018). c-MYC inactivation leads to tumor regression associated with re-differentiation of tumor cells in transgenic mouse models, which confirmed that the block of cell differentiation was one of the activities of c-MYC (Mata-Greenwood et al., 2002; Luo et al., 2019). Our present study showed that the KIM_MYC_AMPLIFICATION_TARGETS_DN signaling pathway was associated with the decrease of c-MYC in NB4 cells treated with JOA. The decrease of c-MYC was verified by real-time PCR and Western blotting analysis. Moreover, G-CSFR is a member of the hemopoietic growth factor receptor family, which mediated the differentiation signal into cells (Fukunaga et al., 1993; Betsuyaku et al., 1999; Maun et al., 2004). As shown earlier, the mRNA and protein expression levels of G-CSFR were significantly upregulated, indicating differentiation of NB4 cells incubated with JOA, as evidenced by the change of cell morphology and increase in the expression of the differentiation antigens CD11b, CD13, and CD14. Therefore, JOA-induced differentiation of NB4 cells may be related to the inhibition of c-MYC.

It is well known that cell cycle arrest is associated with cell differentiation (Hass et al., 1992). CDKN2A and CCND1 play important roles in regulating the transition of cells from the G1 to S phase (Bortolotto et al., 2000; Ma and Li, 2020). It has been shown that upregulation of CDKN2A or downregulation of CCND1 mediated and maintained cell cycle exit at the G1 phase (Gendelman et al., 2017). Our results were in accordance with these findings that JOA induced G0/G1 cell cycle exit in Kasumi-1, KG-1, MUTZ-8, NB4, and HL-60 cells, which is characterized by the increase in the expression of CDKN2A in Kasumi-1 cells and decrease in the expression of CCND1 in NB4 cells at both transcription and protein levels (Figures 7A–C).

In conclusion, our study revealed that JOA inhibited cell proliferation of the M2 subtype of AML cells, especially t(8;21), M3 subtype of AML cells, and leukemic stem-like cells by inducing cell differentiation coupled with cell cycle arrest at the G0/G1 phase. The anti-leukemic activity of JOA is stronger than that of other ent-kaurane diterpenoid compounds such as OGP46. The mechanism of JOA-induced cell differentiation depends on the specificity of the cell type. The effect of JOA on the Kasumi-1 cells was involved in activating the hematopoietic cell lineage signaling pathway, while inhibition of c-MYC was related to the differentiation of NB4 cells induced by JOA. Moreover, JOA can induce differentiation and inhibit the proliferation of leukemic stem-like cells *in vivo*.

Combination chemotherapy based on Ara-C leads to most patients with AML achieving a clinical complete remission (CR). However, approximately 50% of patients achieve CR relapse within 5 years of the date of the diagnosis. This relapse of the disease is considered to be caused by chemotherapy-resistant LSCs (Ma et al., 2021). Herein, the surmounting of the differentiation block by JOA in leukemic stem-like cells and M2 and M3 subtype AML cells may offer a novel therapeutic strategy for the treatment of AML, demonstrating the therapeutic potential to overcome relapse and drug resistance in patients with AML. In addition, because JOA is a promising differentiation-induced agent in patients with AML, mass spectrum analysis in the cell assay or PK studies of JOA merits further investigation.

## Data Availability

The datasets presented in this study can be found in online repositories. The names of the repository/repositories and accession number(s) can be found as follows: NCBI Sequence Read Archive (SRA) database, GSE210200.
